# Knowledge of stroke a study from a sex perspective

**DOI:** 10.1186/s13104-015-1582-1

**Published:** 2015-10-24

**Authors:** José M. Ramírez-Moreno, Rafael Alonso-González, Diego Peral-Pacheco, María Victoria Millán-Núñez, José J. Aguirre-Sánchez

**Affiliations:** Department of Neurology, Hospital Universitario Infanta Cristina, Avda de Elvas s/n, 06080 Badajoz, Spain; Department of Biomedical Sciences, University of Extremadura, Badajoz, Spain; Adult Congenital Heart Centre and Centre for Pulmonary Hypertension, Royal Brompton Hospital, London, UK; Department of History and Bioethics, University of Extremadura, Badajoz, Spain; Department of Cardiology, Hospital Universitario Infanta Cristina, Badajoz, Spain

**Keywords:** Gender, Stroke, Knowledge, Warning signs, Risk factors, Education, Population survey, Women

## Abstract

**Background:**

Public health is increasingly concerned with recognising factors that lead to sex differences in stroke. We conducted a study to determine the effect of sex on knowledge of stroke risk factors and warning signs, and how both are perceived, in a representative sample of adults.

**Methods:**

A representative sample of the population of Extremadura, Spain was selected using a double randomisation technique. Previously trained medical students carried out face-to-face interviews using a structured questionnaire.

**Results:**

2409 subjects were interviewed [59.9 % women; mean age (SD) 49.0 (18.7) years]. Seventy-three percent of all subjects reported at least one correct warning sign of stroke (OR: 1.01; 95 % CI: 0.84–1.21). The most frequently mentioned warning signs were sudden weakness, dizziness, and headache. There were no sex differences regarding the types of warning symptoms that respondents listed. Women displayed better knowledge of risk factors than men (OR: 1.23; 95 % CI: 1.05–1.46). Women were more likely to name hypertension as a risk factor for stroke whereas men more frequently listed smoking, alcohol consumption and a sedentary lifestyle as risk factors. In response to stroke, women were significantly less likely than men to choose to call an ambulance or to go immediately to hospital (OR: 0.69; 95 % CI: 0.60–0.85).

**Conclusions:**

Stroke knowledge is suboptimal in both men and women. We detected better knowledge of stroke risk factors in women, as well as differences in the type of risk factors listed by men and women. There were significant sex differences regarding response to stroke or to its warning signs.

**Electronic supplementary material:**

The online version of this article (doi:10.1186/s13104-015-1582-1) contains supplementary material, which is available to authorized users.

## Background

Prompt recognition of stroke symptoms is critical to timely treatment and women have increased delay to treatment [1]. It is known that stroke is more harmful to women in terms of mortality, functional prognosis, and impact on quality of life. Some studies have shown that women delay going to hospital significantly compared to men, and that they are attended later than men after reaching the hospital [2, 3]. Plausible explanations for these delays are unclear, although they may be due in part to sex-related differences in clinical presentation [4] and also to differences in patients’ knowledge of stroke and response to symptoms.

Numerous studies carried out in the past few years have evaluated knowledge of stroke in the general population, but far fewer of them have analysed sex differences, especially in Europe [5–11]. According to available data, the population’s general knowledge of stroke, its risk factors and warning signs, and response in the event of stroke or stroke symptoms show room for improvement [12]. Limited knowledge can contribute to delays in seeking medical attention after stroke onset, which may have repercussions on the final outcome [13].

The aim of the present study is to identify any sex differences in knowledge of stroke, its vascular risk factors and warning symptoms, illness perception, and attitude towards strokes in a representative sample of adults in the general population.

## Methods

The sample included 2409 subjects older than 18, randomly selected and surveyed with face-to-face interviews. The census districts in Extremadura provided the information used in participant selection. District information is available in the database kept by Spain’s national statistics institute (INE) and our study used data corresponding to January 1, 2008.

The survey instrument was a structured questionnaire divided into four sections with open-and closed-ended questions. The first section of the questionnaire collected information on sociodemographic variables. The second section contained a list of open-ended questions about knowledge of stroke, its symptoms and risk factors, and unhealthy habits. The third section consisted of open-ended questions about the respondent’s hypothetical response to presenting or witnessing signs of a cerebrovascular event, and upon suspecting stroke or transient ischaemic attack (TIA) in a family member or himself/herself. The fourth section contained questions about the respondent’s experiences with the disease and presence of any risk factors or unhealthy habits. The study was approved by the hospital ethics committee. A copy of the final questionnaire may be requested from the corresponding author. A summary of the questionnaire as Additional file 1 is included.

The procedure for collecting information was face-to-face interview. Interviews were conducted by medical students at the University of Extremadura who had previously been trained in the interview method by the research group. Equal numbers of interviews were assigned to each population unit. For each population unit, researchers extracted a second random sample; this one was neighbourhood-specific and every neighbourhood had the same likelihood of being selected. This process yielded a selected street and an alternate street. Each interviewer started at the first house on the list and interviewed the individual who opened the door if that person met eligibility criteria.

Eligible candidates were individuals older than 18 residing in Extremadura who had no cognitive impairment and were willing to participate in the study. All participants gave their express permission to participate. The subject received a copy of the signed and dated consent form.

### Analysis

Data analyses were performed using SPSS software version 15.0 (SPSS Inc.). Some of the variables were recoded as discrete variables to facilitate logistic regression analysis. Knowledge of warning symptoms and classic risk factors was encoded as naming one or more appropriate responses, or none. The dependent variable “hypothetical response to warning symptoms or stroke” was categorised as appropriate (going immediately to hospital or calling the emergency number) or inappropriate (all other options). For purpose of this analysis, subjects were classified by age as younger than 65 or 65 and older. The total sample was also divided into deciles in order to evaluate knowledge by age groups.

We completed a sex-based logistic regression analysis to examine demographic and socioeconomic variables, previous experience with the disease, and presence of vascular risk factors.

## Results

### Sample profile

The final sample comprised 967 men (40.1 %) and 1442 women (59.9 %). There were no significant differences with regard to age or area of residence (Table 1).

**Table 1:** Sociodemographic features and self-reported life-style and medical conditions related to stroke in the study sample by gender

	Men n = 967		Women n = 1442		p
Age in years, median, IQR	48	31.8	49	28.5	0.082
Rural residence, n %	246	25.4	381	26.4	0.587
Education level, n %					0.001
Tertiary	265	27.9	330	23.3	
Secondary	259	27.3	335	23.6	
Primary	304	32.0	507	35.8	
No studies	121	21.8	245	17.3	
Prefer not to answer	18	1.9	25	1.7	
Income €/year, n %					<0.001
≤30,000	606	62.6	886	61.5	
>30,000	91	9.4	45	3.1	
Prefer not to answer	270	27.9	511	35.4	
Occupational status, n %					<0.001
Employed full-time	503	52.0	632	43.9	
Unemployed	102	10.5	307	21.3	
Others	355	36.7	402	28.0	
Prefer not to answer	7	0.7	99	6.9	
Vascular risk factor, n %					
Hypertension	231	23.9	339	23.5	0.795
Dyslipidaemia	261	27.0	304	21.1	0.001
Diabetes	90	9.3	138	9.6	0.892
Smoking	400	41.4	372	25.8	0.010
Alcohol	252	26.1	65	4.5	0.001
Moderate/morbid obesity	168	17.5	119	13.7	<0.001

Thirty-three men and 49 women (3.4 % of each group) reported having suffered a stroke and were able to certify it with medical records. Mean age was 65.1 in females and 68.2 in males. A similar number of respondents stated that a first degree-relative had suffered cerebrovascular disease by sex: 33.4 % of the men and 33.7 % of the women.

### Knowledge of stroke

More women (85.6 vs 82.6 %; *p* = 0.038) knew what cerebrovascular diseases are. And, more women than men (44.3 vs 39.0 %; *p* = 0.035) were familiar with the term “ictus”, the term approved in Spain to denote cerebrovascular event. Most respondents identified the brain as the organ affected by stroke: 70.8 % of men and 72.2 % of women (p = 0.284). However, 15.1 % of men and 13.6 % of women erroneously named the heart (p = 0.324).

### Knowledge of stroke warning signs (SWS)

Knowledge of SWS was tested with open-ended questions and data were later recoded for analysis in logical categories. Respondents able to correctly name at least one warning sign of the disease accounted for 73.4 % of the men and 73.6 % of the women. The mean number of listed symptoms was similar between the sexes: 1.42 (SD: 1.2) for men and 1.43 (SD: 1.2) for women (*P* = 0.871). Likewise, there were no significant sex differences for the type of warning signs that were listed. The most commonly reported stroke symptoms were paralysis/loss of strength, dizziness/instability, and headache. Other warning signs, such as language impairment and vision or sensory disturbances, were reported by less than 1 out of every 8 respondents regardless of sex (Table 2). Facial paralysis was not named as a SWS by any of the respondents. Non-neurological symptoms, such as chest pain or difficulty breathing, were reported by 13.8 % of the men and 13.2 % of the women.

**Table 2:** Stroke warning signs and risk factors reported by survey respondents answering open-ended questions

	Men			Women			p
	n	%	95 % CI^a^	n	%	95 % CI^a^	
Signs and symptoms							
Paralysis/weakness	302	31.8	28.9–34.8	443	30.7	28.4–33–1	0.790
Dizziness/lack of balance	301	31.2	28.3–34.1	439	30.4	28.1–32.8	0.721
Headache	288	29.8	26.9–32.7	444	30.7	28.4–33.2	0.528
Altered consciousness	151	15.6	13.4–18.0	219	15.2	13.4–17.1	0.775
Sudden difficulty speaking	105	10.8	9.1–13.0	185	12.8	11.2–14.7	0.145
Blurred vision, loss of vision	96	9.9	8.2–12.0	146	10.1	8.7–11.8	0.874
Numbness, dead sensation	72	7.4	5.9–9.2	110	7.6	6.4–9.1	0.867
Confusion/disorientation	54	5.6	4.3–7–2	79	5.5	4.4–6–7	0.911
None of the above	195	20.2	17.8–22.8	295	20.5	18.5–22.6	0.861
No neurological symptoms	133	13.8	11.8–16–1	191	13.2	11.6–15–1	0.720
Risk factors							
Smoking	519	53.7	50.5–56.8	696	48.3	45.7–50.8	0.009
Unhealthy diet	393	40.6	37.6–43.8	623	43.2	40.7–45.8	0.211
High blood pressure	320	33.1	30.2–36.1	584	40.5	37.9–43.1	0.002
Alcohol	460	47.6	44.3–50.7	583	40.3	37.9–42.9	0.005
Lack of exercise/unhealthy lifestyle	313	32.4	29.5–35.4	386	26.7	24.5–29.1	0.002
High cholesterol	244	25.2	22.6–28.9	361	25.0	22.8–27.3	0.912
Heart disease	174	17.9	15.7–20.5	280	19.4	17.5–21.5	0.381
Stress	155	16.0	13.8–18.5	256	17.8	15.8–19.8	0.270
Diabetes	129	13.3	11.3–15.6	213	14.8	13.0–16.7	0.323
Obesity	145	14.9	12.8–17.4	190	13.2	11.5–15.0	0.206
Dementia	30	3.1	2.2–4.4	53	3.7	2.8–4.7	0.449
Atherosclerosis	21	2.1	1.4–3.3	26	1.8	1.2–2.6	0.521
Atrial fibrillation	16	1.6	1.0–2.7	18	1.2	0.8–1.9	0.407

Breakdown by sex

*95* *% CI* 95 % confidence interval for proportions

^a^Exact method

**Fig. 1:**
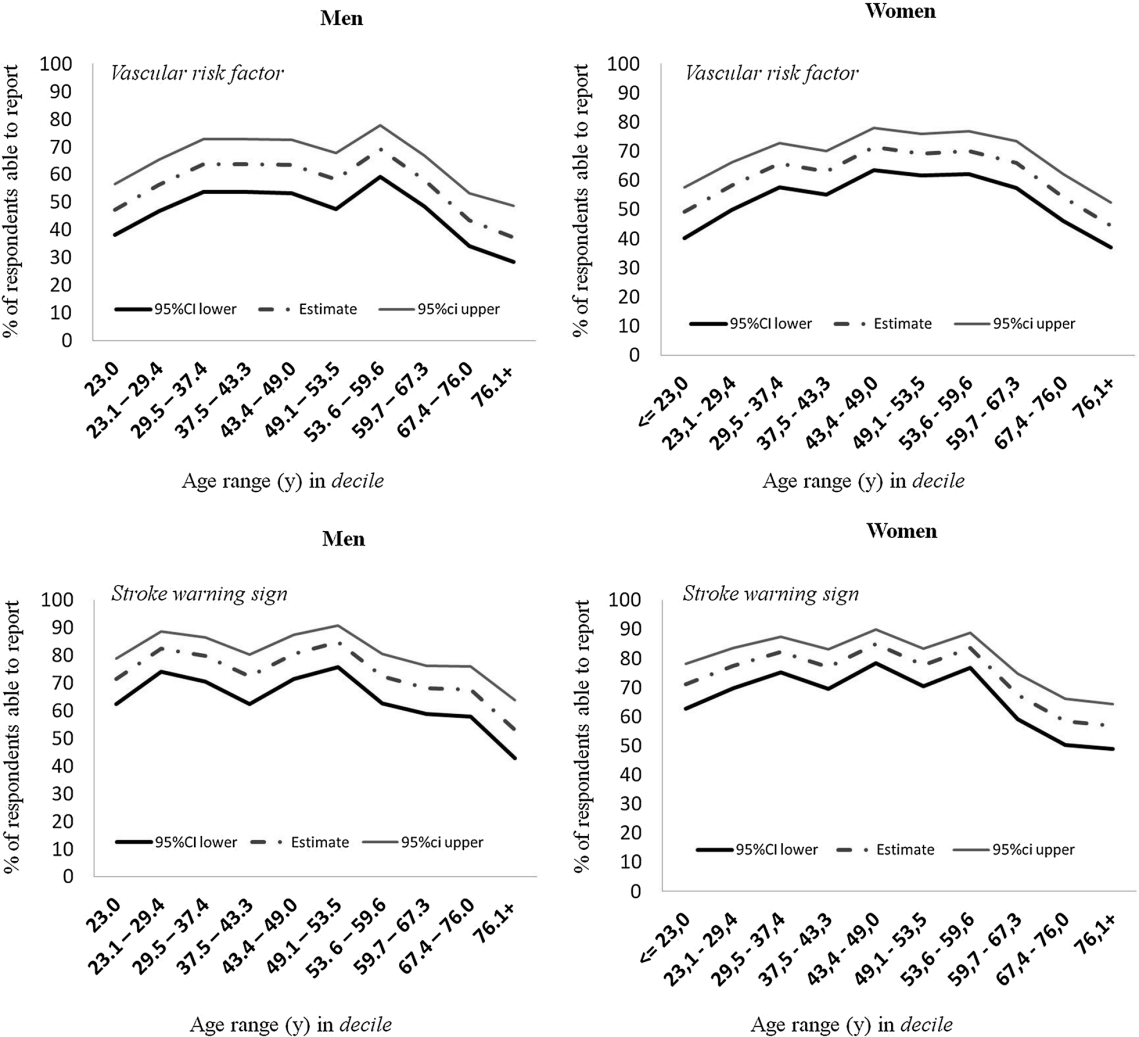
Percentage of respondents able to report at least one stroke warning sign or vascular risk factor broken down by sex and age

Among respondents older than 65, both men (OR: 0.5; 95 % CI: 0.4–0.6) and women (OR: 0.4; 95 % CI: 0.3–0.5) were less likely to name at least one warning sign of stroke. Figure 1 shows that knowledge of at least one SWS, broken down by age, follows an inverted U distribution, with fewer individuals among both the youngest and oldest respondents demonstrating knowledge of stroke.

Area of residence did not significantly influence ability to cite more than one warning symptom, whereas knowledge of symptoms showed a clear and proportional increase with higher educational or income levels. Women with high blood pressure had a significantly better knowledge of SWS (OR: 1.7; 95 % CI: 1.3–2.3). This was also applicable to people with diabetes, regardless of sex. Other participants with vascular risk factors did not display better knowledge; in fact, rates were significantly worse in at-risk groups such as obese men (OR: 0.5; 95 % CI :0.4–0.8), overweight women (OR: 0.6; 95 % CI: 0.5–0.8), obese women (OR: 0.6; 95 % CI: 0.4–0.8) and women with dyslipidaemia (OR: 0.7; 95 % CI: 0.5–0.9). Personal or family experience with the disease was only associated with better knowledge of SWS in women (OR: 1.5; 95 % CI: 1.1–1.9) (Table 3, part 1).

**Table 3:** Sex differences in stroke knowledge broken down by self-reported risk factors and experience with stroke

	Men			Women		
	OR	95 % CI	*P*	OR	95 % CI	*P*
Part 1. Knowledge of at least one warning sign						
Vascular risk factor						
Age > 65 years	0.5	0.4–0.6	<0.0001	0.4	0.3–0.5	<0.0001
Hypertension	1.2	0.8–1.6	0.357	1.7	1.3–2.3	<0.0001
Diabetes	1.6	1.0–2.6	0.029	1.5	1.0–2.2	0.032
Dyslipidaemia	1.3	0.9–1.8	0.128	0.7	0.5–0.9	0.006
Smoking	0.9	0.7–1.3	0.859	0.8	0.6–1.1	0.052
Overweight	1.2	0.8–1.7	0.277	0.6	0.5–0.8	0.001
Obesity	0.5	0.4–0.8	0.001	0.6	0.4–0.8	0.002
Stroke experience						
Personal	2.1	0.8–5.4	0.139	1.2	0.6–2.5	0.524
Relatives	1.3	0.9–1.8	0.740	1.5	1.1–1.9	0.003
Part 2. Knowledge of at least one risk factor						
Vascular risk factor						
Age > 65 years	0.5	0.4–0.6	<0.0001	0.5	0.4–0.7	<0.0001
Hypertension	0.6	0.5–0.9	0.003	1.1	0.8–1.4	0.539
Diabetes	0.9	0.6–1.5	0.825	1.1	0.7–1.5	0.788
Dyslipidaemia	1.4	1.1–2.0	0.010	0.7	0.5–0.9	0.007
Smoking	1.2	0.9–1.5	0.172	0.8	0.7–1.1	0.207
Overweight	0.9	0.7–1.3	0.947	0.9	0.7–1.1	0.865
Obesity	0.7	0.5–1.0	0.684	0.7	0.5–0.9	0.680
Stroke experience						
Personal	0.9	0.5–1.8	0.855	0.8	0.5–1.5	0.548
Relatives	1.5	1.1–1.9	0.008	1.8	1.4–2.4	<0.0001

Univariate analysis

*95* *% CI* 95 % confidence interval, *OR* Odds Ratio

### Knowledge of stroke risk factors

Open-ended questions were also used to test knowledge of stroke risk factors. Respondents able to name at least one vascular risk factor accounted for 61.2 % of women and 56.1 % of men. Women were more likely than men to name one or more risk factors (OR: 1.23; 95 % CI: 1.05–1.46). The risk factors most frequently named by both sexes were tobacco, alcohol, poor diet, high blood pressure, and sedentary lifestyle. High cholesterol level was named by 1 in 4 respondents, diabetes by approximately 1 in 8, and atrial fibrillation by 1/100 respondents (Table 2). Men were more likely to name tobacco (OR: 1.24; 95 % CI: 1.05–1.46), alcohol (OR: 1.33; 95 % CI: 1.13–1.60) and sedentary lifestyle (OR: 1.31; 95 % CI: 1.10–1.56), whereas women were significantly more likely to name high blood pressure, the main risk factor for stroke (OR: 0.72; 95 % CI: 0.61–0.86).

As with knowledge of warning signs, respondents older than 65 were less able to name at least one classic risk factor, this was true of both men and women. Figure 1 shows that knowledge of at least one risk factor broken down by age group follows an inverted U-shaped distribution.

Residents of rural areas were significantly less likely than urban residents to name more than one risk factor. Educational and income levels also exerted a clear and proportional effect. Interestingly, respondents with vascular risk factors did not display a better knowledge, except for men with dyslipidaemia (OR: 1.40; 95 % CI: 1.11–2.00). A prior history of stroke was not associated with better knowledge of risk factors. However, having witnessed the disease in one’s family was linked to better knowledge of stroke (Table 3, part 2).

### Response to stroke

Respondents were asked open-ended questions with no clues about their hypothetical response to experiencing different symptoms suggesting stroke. Next, they were asked how they would respond if they believed they were experiencing a stroke or a TIA. Symptoms causing the most concern in the general population were alterations of consciousness, sudden onset of weakness, and difficulty speaking; visual disturbances and intense headache caused the least concern. Significantly more men than women selected an appropriate response for all listed symptoms: alterations of consciousness (OR: 1.44; 95 % CI: 1.18–1.76), sudden weakness (OR: 1.33; 95 % CI: 1.11–1.60), language impairment (OR: 1.35; 95 % CI: 1.14–1.60), visual disturbances (OR: 1.40; 95 % CI: 1.10–1.65) and headache (OR: 1.27; 95 % CI: 1.08–1.50). Regardless of sex, we observed that appropriate response to all symptoms was clearly less frequent among older respondents.

An appropriate response to a suspected stroke was indicated by 83.4 % of men and 77.5 % of women. The difference between men’s and women’s response to stroke was statistically significant (OR: 1.45; 95 % CI: 1.18–1.79), and it remained after adjusting for age, area of residence, educational level and income level (OR: 1.32; 95 % CI: 1.01–1.73). Responses given by participants with vascular risk factors were poor overall, and only female smokers indicated more appropriate responses to suspected stroke (OR: 1.86; 95 % CI: 1.36–2.55). Rates of appropriate responses in women with hypertension, and in men with hypertension, diabetes, and dyslipidaemia, were significantly lower than in subjects without these risk factors. Respondents with a history of stroke were not more likely to respond appropriately in the event of an additional stroke. In fact, this group’s rate of correct responses was significantly lower than that of the rest of the population in both sexes: men (OR: 0.38; 95 % CI: 0.18–0.79) and women (OR: 0.31; 95 % CI: 0.17–0.55). Experience with the disease in the family was not associated with higher rates of appropriate responses to suspected stroke in either men (*p* = 0.190) or women (*p* = 0.317).

**Fig. 2:**
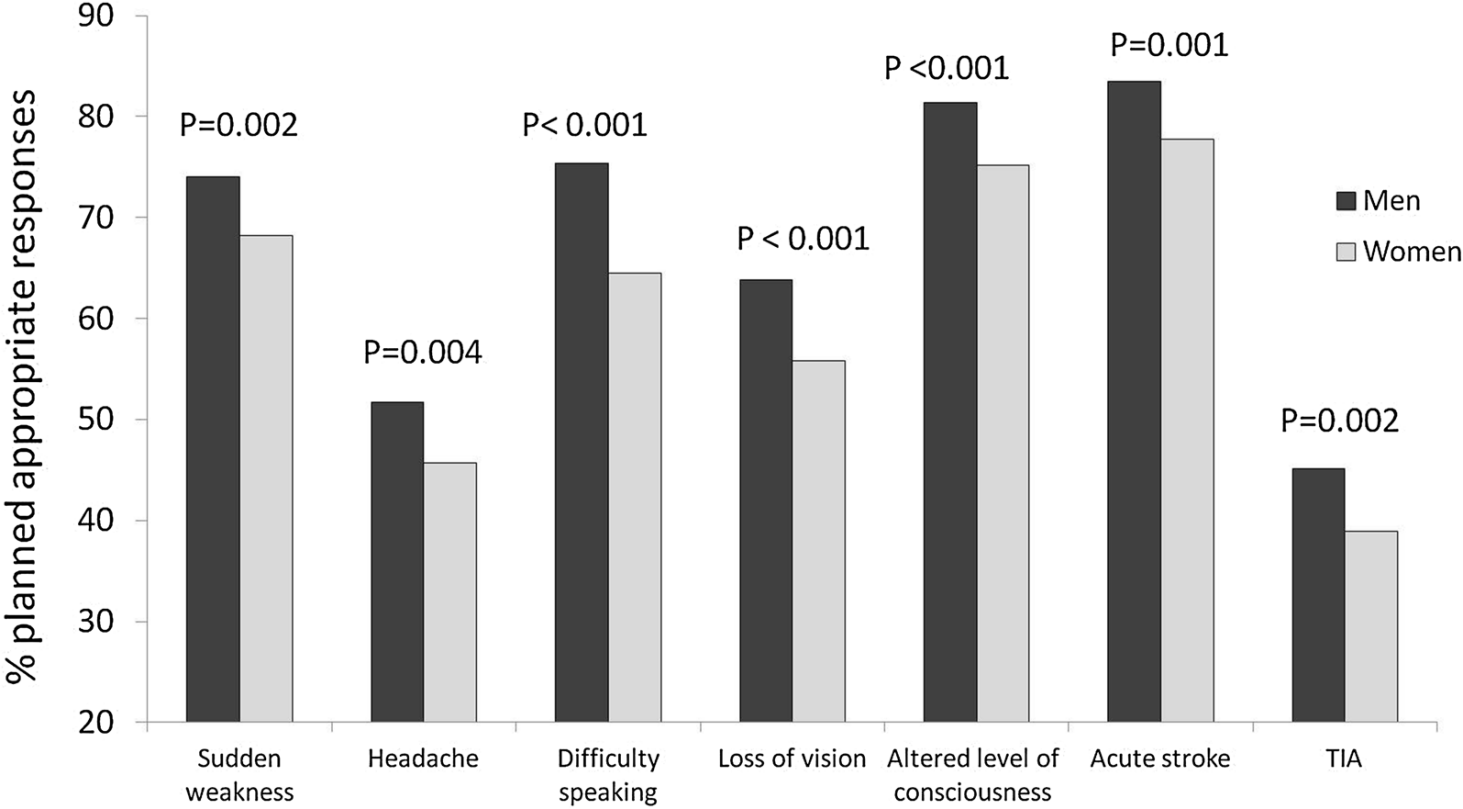
Percentage of appropriate responses planned in the event of acute stroke, TIA, or listed stroke symptoms. Breakdown by sex

Upon suspecting a TIA, men would be more likely than women to go immediately to the hospital or call the emergency number (45.1 vs 38.8 %). These differences were found to be significant in the univariate analysis (OR: 1.29; 95 % CI: 1.10–1.52), but they were no longer significant after adjusting for age, area of residence, educational level and income level (*p* = 0.449) (Fig. 2).

## Discussion

Stroke knowledge has been addressed by many population-based cross-sectional studies, although few of them have investigated sex effects [14].

Previous studies have defined knowledge of cerebrovascular disease symptoms as the ability to name one or more warning signs in response to open-ended questions [15]. Our study found no significant sex differences in the number of correctly identified SWS or the type of signs mentioned. Most of the studies conducted in Western countries have shown either that women are more able to name warning signs of stroke [9, 16–21] or that no significant sex differences are present [6, 22, 23]. A study conducted more than 10 years ago in Spain showed that only 32.6 % of the population was able to identify at least one SWS and that men were considerably more knowledgeable about stroke than women [5]. However, as confirmed by our study, the situation in Spain has clearly changed over time. More than 70 % of this population is able to name at least one symptom of stroke. As reported by other studies [6, 8, 9, 18], the most frequently named warning symptoms were paralysis, dizziness, and headache. Approximately 1 out of every 8 individuals wrongly identified chest pain and shortness of breath as SWS; this reflects the confusion between ischaemic heart disease and stroke in part of the general population [15, 17]. The factors associated with a better knowledge of stroke symptoms are being younger than 65, higher educational level, and higher income level. Similar findings have been published in earlier studies [5, 12]. Other studies have also reported that individuals with a history of stroke or vascular risk factors do not display more ample knowledge of the disease [18, 22, 24]. These findings have important implications for health education, since this population is at the most risk for stroke. In our study, only women with high blood pressure and individuals with diabetes seemed to be better informed.

Women in our population display considerably more knowledge of risk factors than men, which coincides with findings from other studies [15–17]. Few studies have shown a greater knowledge of stroke risk factors among men [5]. There are differences between the types of vascular risk factors listed by men and women. While men more frequently named risk factors associated with lifestyle, women were more likely to cite medical conditions such as high blood pressure. A possible explanation for this tendency is that women seem to be better informed about disease prevention than men, and they may also be more interested in health-related issues [14].

Few studies have examined sex differences in response to stroke [5, 9, 23, 25, 27]. Concerning immediate response to stroke or its warning signs, men are significantly more likely than women to call an ambulance or go immediately to hospital. These differences remain after adjusting for age, residence or socioeconomic level, factors shown by previous research to be associated with a more appropriate response to stroke [20, 25–27]. These findings are especially relevant since some studies reveal that women tend to delay going to hospital more than men [2, 3, 28]. Prehospital delay is known to be one of the major factors limiting use of reperfusion therapy for stroke because stroke has a narrow therapeutic window [29]. A meta-analysis showed that thrombolytic treatment was more effective in women than in men. However, proportionally fewer women than men do not receive tPA after acute stroke [30]. Fewer women than men reach hospital within the first hours after symptom onset, and this observation may partially explain why women are less likely to be treated with thrombolysis [4]. There is no reasonable explanation for this tendency, although we suggest that men and women may not perceive the severity of the disease in the same way, and women may underestimate the urgency of warning signs. Some studies have shown that while women are aware that cardiovascular disease is one of the main causes of death, only a small percentage of them regard ischaemic heart disease and stroke as the greatest threat to their health [29]. This misperception may be one of the obstacles to women who require medical attention.

Our study has some methodological limitations. First, the survey may have a selection bias since the numbers of male and female participants did not match exactly. Second, there may also be a non-response bias, given that individuals who refused to participate or could not be contacted might have a different level of stroke knowledge. Third, results depend on the type of questionnaire; in face-to-face interviews, the interviewer may unintentionally influence the results and lead to inconsistent measurements. Lastly, cross-sectional studies cannot prove causality.

A number of tests have been developed to assess specific stroke knowledge, including the stroke symptom questionnaire, stroke action test, and stroke awareness questionnaire, in which residents learn how to dial 911, etc., for general directions when a stroke has occurred [31]. However, there are presently few references to methods assessing community resident knowledge regarding ways to cope with pre-hospital stroke symptoms in the scientific literature. Therefore, other limitation of this job is that no psychometric properties that show the validity and reliability of this type of survey.

## Conclusions

Severity of stroke symptoms, mortality rates, and residual disability rates are higher in women, which results in lower quality of life after stroke [1, 2]. Stroke’s differential effect on women will continue to gain importance in the decades to come. Therefore, public awareness campaigns must be implemented to increase knowledge of stroke in the general population and make women aware of the importance of going immediately to hospital if they experience warning signs of stroke. Both the general population and the scientific and medical communities stand to benefit from better education on stroke.

## Additional file

10.1186/s13104-015-1582-1 Description of the survey instrument.

## Abbreviations

SD: standard deviation
OR: odds ratio
CI: confidence interval
TIA: transient ischaemic attack
SWS: stroke warning signs
tPA: tissue plasminogen activator
