# Supplementary material for: Knowledge of stroke a study from a sex perspective
Source: BMC Res Notes. 2015 Oct 24;8:604. doi: 10.1186/s13104-015-1582-1 (PMC4620012; doi:10.1186/s13104-015-1582-1)
Supplement: Supplementary file 1 — 10.1186/s13104-015-1582-1 Description of the survey instrument. [file 13104_2015_1582_MOESM1_ESM.docx]

The survey instrument was a questionnaire designed to assess information about stroke. It was pretested using a sample of 33 individuals. Based on results from the pre-test, some wording was changed to improve question clarity. A copy of the final questionnaire (in Spanish) may be requested from the corresponding author.

The final questionnaire contained 33 questions divided into 4 sections.

The first section collected information about the following sociodemographic factors: sex, age, rural or urban area of residence (population < 10 000 or ≥ 10 000 respectively), educational level (no studies or incomplete primary studies, primary, secondary, or tertiary), income level (< 10 000, 10 001–20 000, 20 001–30 000, 30 001–40 000, or > 40 000 euros/year), marital status (single, married, widowed, separated, divorced), professional status (student, employed full-time, unemployed, retired), self-rated health status (excellent, good, fair, poor).

The second section contained a series of open-ended questions about stroke, its symptoms, risk factors, and unhealthy habits. Symptoms considered valid were all neurological warning symptoms similar to intense headache, paralysis, sensory disturbances, vision disturbances, loss of balance, language or speech impairment, or loss of consciousness. Classic risk factors for developing stroke were considered valid: age, hypertension, diabetes mellitus, hypercholesterolemia, atrial fibrillation, arteriosclerosis, and heart disease. Correct answers for unhealthy stroke-promoting habits included smoking, alcohol abuse, drug consumption, sedentary lifestyle, obesity, and high levels of stress. This section also contained closed-ended and specific questions about the implications of each individual risk factor and unhealthy habit.

The third section of the questionnaire consisted of an open-ended question regarding the respondent’s hypothetical reaction upon identifying signs of a stroke or transient ischemic attack in a family member or himself/herself. For purposes of analysis, these answers were codified as seven possibilities: calling the general practitioner (GP) or family physician (FP), going to the GP or FP’s office, going directly to the hospital, calling the emergency telephone number (112), waiting and then seeking medical assistance, doing nothing, or don’t know. The questionnaire included questions about the individual’s perception of stroke severity and how stroke severity compares to that of other vascular diseases, such as acute myocardial infarction. Participants were also asked about their sources for information on stroke.

The fourth section inquired about the individual’s experience with the disease and any risk factors or unhealthy habits. Here, participants were specifically asked if they had a personal or direct family history of stroke, and if they presented any of the classic cerebrovascular risk factors (smoking habit and number of cigarettes per day, diagnosis of hypertension or use of medication specifically intended to decrease blood pressure, diagnosis of diabetes or use of antidiabetic drugs, having elevated cholesterol levels or taking statins, or drinking two or more glasses of wine, beer, or other alcoholic beverages daily). Each participant’s body mass index was calculated by weight and height.

Key Themes Emerging from Survey

1. Sociodemographic variables: gender, age, place of residence, education level, socioeconomic status, marital status, employment status and health status
2. Knowledge word “ictus", stroke, TIA and warning signs
3. Knowledge of associated cardiovascular risk factors and related lifestyle
4. Hypothetical attitude to symptoms individually and stroke or TIA
5. Gravity perception
6. Personal or family experience with illness
7. Presence of risk factors and harmful lifestyles
8. Sources of information used by the population
